# Reconnecting groups of space debris to their parent body through proper elements

**DOI:** 10.1038/s41598-021-02010-x

**Published:** 2021-11-22

**Authors:** Alessandra Celletti, Giuseppe Pucacco, Tudor Vartolomei

**Affiliations:** 1 Department of Mathematics, University of Rome Tor Vergata, Via della Ricerca Scientifica 1, 00133 Rome, Italy; 2 Department of Physics, University of Rome Tor Vergata, Via della Ricerca Scientifica 1, 00133 Rome, Italy

**Keywords:** Applied mathematics, Scientific data

## Abstract

Satellite collisions or fragmentations generate a huge number of space debris; over time, the fragments might get dispersed, making it difficult to associate them to the configuration at break-up. In this work, we present a procedure to back-trace the debris, reconnecting them to their original configuration. To this end, we compute the proper elements, namely dynamical quantities which stay nearly constant over time. While the osculating elements might spread and lose connection with the values at break-up, the proper elements, which have been already successfully used to identify asteroid families, retain the dynamical features of the original configuration. We show the efficacy of the procedure, based on a hierarchical implementation of perturbation theory, by analyzing the following four different case studies associated to satellites that underwent a catastrophic event: Ariane 44lp, Atlas V Centaur, CZ-3, Titan IIIc Transtage. The link between (initial and final) osculating and proper elements is evaluated through tools of statistical data analysis. The results show that proper elements allow one to reconnect the fragments to their parent body.

## Introduction

Since the launch of Sputnik 1 in 1957, thousands of satellites have been deployed in orbit around the Earth. Explosions or collisions of satellites generated millions of space debris of various sizes^1^, currently traveling at different altitudes: rocket stages, fragments from disintegrations, bolts, paint flakes, electronic parts, etc. Chain reactions triggered by catastrophic events involving satellites might increase the hazard of (human and robotic) space activities. A single break-up event generates a cloud of debris which scatters around, sometimes reaching great distances after a relatively short time. Once the fragments are dispersed, it is often difficult to trace them back; hence, a question of paramount importance is to connect the debris to their parent satellite. In this work we propose a method that allows us to link the fragments, after a certain interval of time, to the configuration of the debris soon after the initial catastrophic event. This result contributes to address a timely problem since, in case of a collision between two satellites or an explosion of a single satellite, it is certainly important to know the parent bodies that generated the space debris. The implications are wide and range from space sustainability to space law.

To study a specific break-up event, we introduce a suitable model (based on the Hamiltonian formalism) to describe the dynamics of each fragment. The model is composed of the sum of the Keplerian attraction, the effect of the geopotential, the gravitational influence of Sun and Moon. Then, we implement perturbation theory to construct a sequence of canonical transformations providing, for each debris, approximate integrals of motion called *proper elements, *namely quantities that stay nearly constant over time. Each fragment is characterized by a set of six orbital elements, namely semimajor axis *a*, eccentricity *e*, inclination *i*, mean anomaly *M*, argument of perigee $\omega$ and longitude of the ascending node $\Omega$. Starting from their initial values, we compute the orbital elements of each fragment after a given interval of time, to which we refer as the *final osculating elements. *Then, we compute the proper elements associated to the final osculating elements, and we compare them either with the initial elements and with the corresponding proper elements at the initial time. The comparison gives the desired information: while the final osculating elements might spread far away from the initial values, the (initial/final) proper elements stay almost constant and retain the original features of the cloud of fragments^2^. A striking use of proper elements was already proposed to group asteroids, inspired by the pioneer work^3^ of Hirayama in 1918 and continued by many other authors^4,5^. The analytical computation of proper elements allowed to group asteroids in families, possibly leading to the conjecture that such asteroids might be fragments of an ancestor parent body. Knežević and Milani^6^ introduced also the synthetic proper elements based upon a numerical integration, a digital filtering of the short-period terms and a Fourier analysis.

Motivated by the successful results on asteroids, we propose to group and reconnect space debris through the computation of the proper elements associated to fragments generated by a satellite break-up event^7–9^. The procedure we are going to describe, requires the introduction of a realistic model which depends on quantities varying on different time scales; hence we need a suitable hierarchical set of transformations of coordinates, called normal forms, aimed at constructing the proper elements, whose relation with the initial elements is analyzed through statistical methods. We will consider four sample cases associated to the break-up events of the satellites Ariane 44lp, Atlas V Centaur, CZ-3, Titan IIIc Transtage. Using statistical data analysis, we show the effectiveness of the use of proper elements in reconnecting the fragments to their parent body. To reconnect the debris to a parent body, we back-propagate the debris for a given time and compare the osculating or proper elements at the initial time and at the back-propagated time. The effectiveness of the method has been shown in the specific example of Titan III Transtage. We finally provide an example in which one can distinguish between proper elements associated to nearby breakup events.

This work is organized as follows. After introducing the model, we describe the procedure to compute the proper elements through normal form theory. Then we investigate the test cases by computing osculating and proper elements, and by analyzing the results through histograms, Kolmogorov-Smirnov test, Variance Equivalence test and Pearson correlation coefficient. We end up with some conclusions and perspectives.

## The model

For the present work, our case studies will be located at altitudes between 15000 and 25000 km, all of them well above the Earth’s atmosphere. At those altitudes a celestial object is subject to different forces that we describe through a Hamiltonian function composed by the following parts: the attraction of the Earth (that we split as the sum of the Keplerian part $H_{Kep}$ and the potential $H_E$ generated by the Earth’s non-spherical shape), and the gravitational influence of the Moon $H_M$ and the Sun $H_S$ (both assumed to be point masses). The overall Hamiltonian

1 $$\begin{aligned} H = H_{Kep} + H_E + H_M + H_S \end{aligned}$$

depends upon the orbital elements of the debris, Moon and Sun, and on the sidereal time describing the rotation of the Earth^10^.

We are aware that a realistic model should include also the effect of the solar radiation pressure^11^ (SRP). However, we decided not to consider SRP for two main reasons: (i) the work^2^ provides some experiments on synthetic space debris (namely obtained through a simulator of break-up events), using a model that includes SRP; however, the results show that at intermediate altitudes the computation of the proper elements is not much affected by SRP, at least for objects with an area-to-mass ratio lower than 0.74; (ii) there does not exist a public catalogue that provides information about the area-to-mass ratio of real space debris, thus preventing reliable experiments on real cases.

### The Keplerian and geopotential Hamiltonians

Expressing the Hamiltonian in terms of the orbital elements, the Keplerian part $H_{Kep}$ is given by

$$\begin{aligned} H_{Kep} = - {{G M_E}\over {2a}}, \end{aligned}$$

where G is the gravitational constant and $M_E$ is the mass of the Earth.

The contribution $H_E$ due to the Earth’s non-spherical shape is computed as follows^12,13^: we expand the geopotential in spherical harmonics, then we average over the fast variables (namely the mean anomaly of the debris and the sidereal time), and finally we limit the expansion of the secular part of the geopotential to the greatest spherical harmonic coefficients, usually denoted as $J_2$ and $J_3$. The resulting Hamiltonian takes the form:

$$\begin{aligned} H_E = G M_E {R_E^2\over a^3} J_2 \left( {3\over 4} \sin ^2 i -{1\over 2}\right) {1\over {(1-e^2)}^{3/2}} + G M_E {R_E^3\over a^4} J_3 \left( {{15}\over {8}} \sin ^3 i -{3\over 2} \sin i\right) e \sin \omega {1\over {(1-e^2)}^{5/2}}\ , \end{aligned}$$

where $R_E$ is the Earth’s radius (equal to 6378.1363 km).

### Moon and Sun Hamiltonians

The Hamiltonians of the Moon $H_M$ and of the Sun $H_S$ are expanded in powers of the ratios of the semimajor axes of the debris, and of the Moon and Sun, respectively $a_M$ and $a_S$, as well as in powers of the eccentricity and of the cosine of the inclination. The resulting expansions are truncated to a low order (typically the second one). By $(a_b,e_b,i_b,M_b,\omega _b,\Omega _b)$ we denote the orbital elements of the third body perturber, where $b=M$ and $b=S$ correspond to the Moon and Sun, respectively.

The expansion of the Moon’s Hamiltonian in terms of the orbital elements of the Moon and the debris is given below; we underline that in applications we will consider the expansion of $H_M$ in (1) to $l=2$:

$$\begin{aligned} H_M= & {} -G m_M \sum \limits _{l\ge 2}\sum \limits _{m=0}^{l}\sum \limits _{p=0}^{l}\sum \limits _{s=0}^{l}\sum \limits _{q=0}^{l} \sum \limits _{j=-\infty }^{\infty }\sum \limits _{r=-\infty }^{\infty } (-1)^{m+s} (-1)^{[m/2]}\nonumber \\&\frac{\epsilon _m \epsilon _s}{2a_M} \frac{(l-s)!}{(l+m)!}\left( \frac{a}{a_M}\right) ^l F_{lmp}(i) F_{lsq}(i_M) H_{lpj}(e) G_{lqr}(e_M)\nonumber \\&\{(-1)^{t(m+s-1)+1}U_l^{m,-s} \cos (\phi _{lmpj}+\phi '_{lsqr}-y_s \pi )\nonumber \\&+ (-1)^{t(m+s)}U_l^{m,-s}\cos (\phi _{lmpj}-\phi '_{lsqr}-y_s \pi )\}\ , \end{aligned}$$

where $m_M$ is the mass of the Moon, $y_s = 0$, if (*s* mod 2)=0, $y_s = \frac{1}{2}$, if (*s* mod 2)= 1, $t = (l-1)$ mod 2, and

$$\begin{aligned} \epsilon _m= & {} {\left\{ \begin{array}{ll} 1,&{} m=0\\ 2,&{} m\in {\mathbb {Z}}\backslash \{0\} \end{array}\right. }\\ \phi _{lmpj}= & {} (l-2p)\omega + (l-2p+j)M+m\Omega \\ \phi '_{lsqr}= & {} (l-2q)\omega _M + (l-2q+r)M_M+s\left( \Omega _M-\frac{\pi }{2}\right) \ . \end{aligned}$$

The functions $F_{lmp}(i)$, $F_{lsq}(i_M)$ and $G_{lqr}(e_M)$ can be found, e.g., in^10,12^; $H_{lpj}(e)$ are the Hansen coefficients, while the terms $U_l^{m,s}$ are given by

$$\begin{aligned} U_l^{m,s} = \sum \limits _{r=\max (0,-(m+s))}^{\min (l-s,l-m)}(-1)^{l-m-r}{l+m\atopwithdelims ()m+s+r}{l-m\atopwithdelims ()r}\cos ^{m+s+2r} \left( {\epsilon \over 2}\right) \sin ^{-m-s+2(l-r)}\left( {\epsilon \over 2}\right) , \end{aligned}$$

where $\epsilon = 23^o26'21.406''$ is the Earth’s obliquity.

The Hamiltonian due to the Sun depends on the orbital elements of the Sun and the debris. The expansion of $H_S$ is given below and, again, we will consider the expansion to $l=2$:

$$\begin{aligned} H_S = -G m_S \sum \limits _{l\ge 2}\sum \limits _{m=0}^{l}\sum \limits _{p=0}^{l}\sum \limits _{h=0}^{l} \sum \limits _{q=-\infty }^{\infty }\sum \limits _{j=-\infty }^{\infty } \frac{a^l}{a_S^{l+1}}\epsilon _m\frac{(l-m)!}{(l+m)!}\ F_{lmp}(i) F_{lmh}(i_S) H_{lpq}(e) G_{lhj}(e_S) \cos (\phi _{lmphqj}), \end{aligned}$$

where $m_S$ is the mass of the Sun and

$$\begin{aligned} \phi _{lmphqj} = (l-2p)\omega + (l-2p+q)M - (l-2h)\omega _S - (l-2h+j)M_S + m(\Omega -\Omega _S). \end{aligned}$$

### Validation of the model

**Figure 1:**
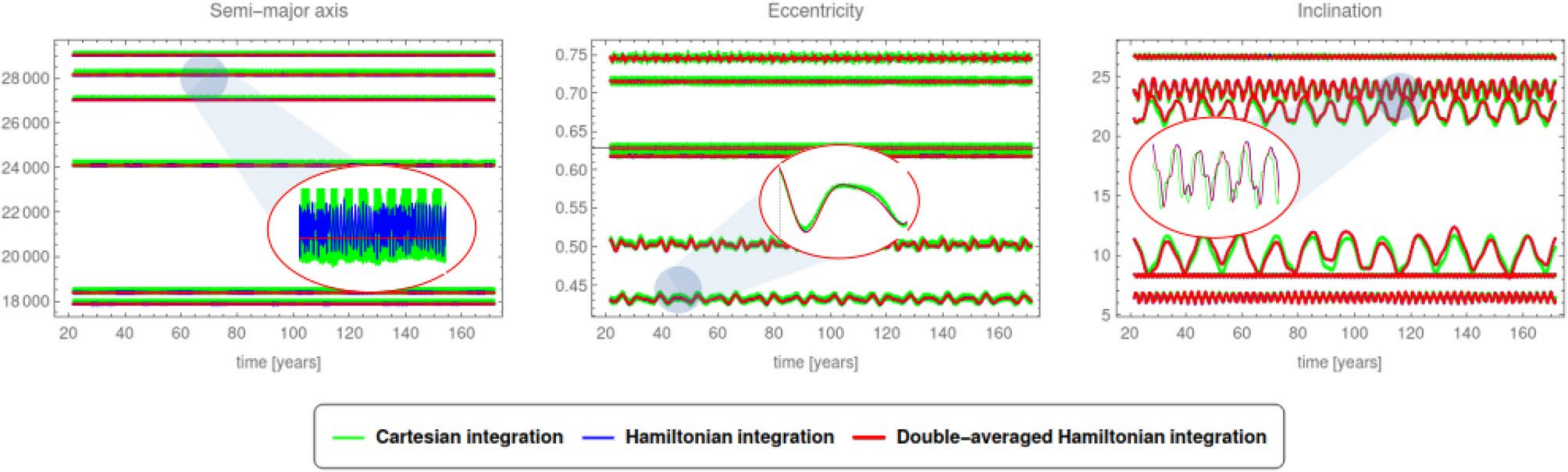
Comparison between the Cartesian integration of the state equations of motion (green line), Hamilton’s equations of the full Hamiltonian *H* (blue line), Hamilton’s equations of the doubly averaged Hamiltonian (red line).

Although representing an approximation of the full model, the Hamiltonian *H* in (1) provides an accurate description of the dynamics, as shown by the comparison in Fig. 1 between the integration of Hamilton’s equations and the Cartesian equations of motion^13^.

Besides depending on the orbital elements of the debris, the Hamiltonian depends also upon the orbital elements of Moon and Sun. For our purposes, it is essential to stress that the debris, Moon and Sun move on different time-scales, since the angular variables describing their respective motions vary with rates of the order of days (for the debris), months (for the Moon), years (for the Sun), see Table 1. As a consequence, the respective angular variables of debris, Moon and Sun can be ordered hierarchically as fast, semi-fast and slow. The fast angles are indeed the mean anomaly of the debris and the sidereal time accounting for the rotation of the Earth; we report in Fig. 1 also the integration obtained using the Hamiltonian, doubly averaged with respect to such fast angles.

**Table 1:** The orbital elements (*M*, *a*, *e*, *i*) and the rates of variations of $(\omega , \Omega )$ of Moon and Sun.

	Moon	Sun
Mean daily motion	13.06°/day	1°/day
Semimajor axis	384478 km	1.496 10^8^ km
Eccentricity	0.0549	0.0167
Inclination	5° 15′	23° 26′ 21.406″ 4
Rate of variation of $\omega$	0.16°/day	282.94°/day
Rate of variation of $\Omega$	− 0.0529918°/day	0°/day

## Normal form and proper elements

We briefly recall the basics of normal form theory^14^, which is at the basis of the computation of the proper elements. We consider a Hamiltonian of the form

2 $$\begin{aligned} {\mathscr {H}}({{\underline{I}}},{{{\underline{\varphi }}}}) = {\mathscr {H}}_0({{\underline{I}}}) + \varepsilon {\mathscr {H}}_1({{\underline{I}}},{{{\underline{\varphi }}}})\ , \end{aligned}$$

where $({{\underline{I}}},{{{\underline{\varphi }}}})$ are action-angle variables with $({{\underline{I}}},{{{\underline{\varphi }}}})\in B\times {{{\mathbb {T}}}}^n$, where $B\subset {{{\mathbb {R}}}}^n$ is an open set and *n* denotes the number of degrees of freedom. In (2) the function ${\mathscr {H}}_0({{\underline{I}}})$ is the integrable part, $\varepsilon \in {{\mathbb {R}}}$ is a small parameter, ${\mathscr {H}}_1({{\underline{I}}},{{{\underline{\varphi }}}})$ is the perturbing function.

The normalization procedure consists in the definition of a suitable change of coordinates that transforms the Hamiltonian, so that it becomes integrable to orders of $\varepsilon ^2$. The procedure can be iterated for some steps, but it is known that in general it is not converging^15^.

We assume that the function ${\mathscr {H}}_1$ can be expanded in Fourier series as

$$\begin{aligned} {\mathscr {H}}_1({{\underline{I}}},{\underline{\varphi }})=\sum _{{{\underline{k}}}\in K} b_{{\underline{k}}}({{\underline{I}}})\exp ( i {{\underline{k}}}\cdot {{{\underline{\varphi }}}})\ , \end{aligned}$$

where $K\subseteq {{{\mathbb {Z}}}}^n$ and $b_{{\underline{k}}}$ are functions with real coefficients. Let $\chi$ be the generating function of the canonical transformation from the variables $({{\underline{I}}},{{{\underline{\varphi }}}})$ to the new variables $({\underline{I}}',{{{\underline{\varphi }}}}')$ given by

$$\begin{aligned} {{\underline{I}}}= S_{\chi }^{\varepsilon }{{\underline{I}}}'\ ,\qquad {\underline{\varphi }} = S_{\chi }^{\varepsilon }{{{\underline{\varphi }}}}'\ , \end{aligned}$$

where the action of $S_{\chi }^{\varepsilon }$ is defined by

$$\begin{aligned} S_{\chi }^{\varepsilon }{\mathscr {F}} := {\mathscr {F}} + \sum _{i=1}^{\infty } \frac{\varepsilon ^i}{i!}\{\{...\{{\mathscr {F}},\chi \}, \dots \},\chi \} \end{aligned}$$

with $\{\cdot ,\cdot \}$ the Poisson bracket operator. We determine $S_{\chi }^{\varepsilon }$ by requiring that the new Hamiltonian ${\mathscr {H}}^{(1)} = S_{\chi }^{\varepsilon }{\mathscr {H}}$ is transformed into

3 $$\begin{aligned} {\mathscr {H}}^{(1)}({{\underline{I}}}',{{{\underline{\varphi }}}}') = Z_1({{\underline{I}}}') + \varepsilon ^2 {\mathscr {H}}_2({{\underline{I}}}',{{{\underline{\varphi }}}}')\ , \end{aligned}$$

where $Z_1={\mathscr {H}}_0+\varepsilon \overline{{\mathscr {H}}}_1$ is integrable ($\overline{{\mathscr {H}}}_1$ is the average of ${\mathscr {H}}_1$) and ${\mathscr {H}}_2$ is the remainder term of order $\varepsilon ^2$. Inserting the change of coordinates in (2), one obtains the transformed Hamiltonian which takes the desired form (3) provided $\chi$ satisfies the following normal form equation:

$$\begin{aligned} {\mathscr {H}}_1({{\underline{I}}}',{{{\underline{\varphi }}}}')+\{ {\mathscr {H}}_0({{\underline{I}}}'), \chi ({{\underline{I}}}',{{{\underline{\varphi }}}}')\} =\overline{{\mathscr {H}}}_1({{\underline{I}}}'). \end{aligned}$$

Expanding $\chi$ in Fourier series, denoting the frequency by ${\underline{\omega }}_0= {{\partial {\mathscr {H}}_0}\over {\partial {{\underline{I}}}'}}$, one obtains that the generating function is given by the following formula, which is valid under the non-resonance assumption ${{\underline{k}}\cdot {{{\underline{\omega }}}}_0}\not =0$:

$$\begin{aligned} \chi ({{\underline{I}}}',{\underline{\varphi }}')=- i \sum _{{\underline{k}}\in K} \frac{b_{{\underline{k}}}({{\underline{I}}}')}{{\underline{k}}\cdot {{{\underline{\omega }}}}_0}\ \exp ( i {\underline{k}}\cdot {{{\underline{\varphi }}}}')\ . \end{aligned}$$

A higher order normal form is obtained by iterating the above procedure.

Recalling that the space debris model described above depends on fast, semi-fast and slow variables, we compute the normal form, taking advantage of the hierarchical structure of the coordinates associated to the debris, Moon and Sun. We first average the Hamiltonian over the fast (mean anomaly of the debris and sidereal time) and semi-fast (mean anomalies of Moon and Sun) angles. According to Hamilton’s equations, the rate of variation of the semimajor axis of the debris is given by the derivative of the Hamiltonian with respect to the mean anomaly; since we averaged over the mean anomaly, the semimajor axis is constant and becomes the first proper element, namely a quasi-integral of motion for the averaged approximated model. After averaging over the mean anomalies and the sidereal time, we end-up with a Hamiltonian function with three degrees-of-freedom in the extended phase space, since the Hamiltonian depends on time through the variation of the longitude of the ascending node of the Moon (see Table 1).

Next, we consider some reference values for the eccentricity and the inclination (namely the values of the fragments of the case study) and we expand the averaged Hamiltonian around such values. Then, we implement a canonical change of variables through a Lie series normalization, implemented through a Mathematica$^{\copyright }$ program, that removes the dependence on the angles; this procedure provides two more proper elements associated with the eccentricity and the inclination. By making explicit all transformations^2^, we end the procedure by back-transforming the change of variables to express the proper elements in the original coordinates.

In conclusion, the procedure leading to the computation of the proper elements can be summarized as follows^2^.

1. We consider the Hamiltonian including the contributions of the gravitational attractions of the Earth, Moon and Sun; we average with respect to the fast variables, in particular the mean anomaly *M*; hence, the semimajor axis is constant and becomes the first proper element.
2. Since the longitude of the ascending node of the Moon $\Omega _M$ depends on time, the Hamiltonian resulting from step 1 depends on $(e,i,\omega ,\Omega ,t)$; hence, we introduce the Hamiltonian in the extended phase space, so that it becomes autonomous, although depending on one more additional variable.
3. We fix reference values for $e_0$ and $i_0$, and we introduce new variables $\eta$ and $\iota$ such that $e=e_0+\eta$, $i=i_0+\iota$.
4. We expand the Hamiltonian in power series around $\eta =0$, $\iota =0$ up to order 3 in $\eta$, $\iota$.
5. We split the resulting Hamiltonian into the linear part and a remainder. We compute the generating function and the canonical transformation of coordinates to remove the remainder to higher orders.
6. Once obtained the new normal form, we disregard the remainder, so that the two actions corresponding to eccentricity and inclination become constants of motion.
7. The initial values of the new constants of motion, which are the two additional proper elements, are obtained back-transforming the canonical transformations in terms of the original variables, namely in terms of the initial data.

For a specific case, we compute the osculating and proper elements by integrating the equations of motion and by computing the normal form using a Mathematica$^{\copyright }$ program. We summarize below the steps of the procedure which will be implemented for each of the fragments of the case studies analyzed in the next sections.

- *Step 1.* INPUT: set the normalization parameters: maxSteps=maximum normalization steps, maxR=number of terms kept in the remainder after each step, maxTaylor=maximum order of the Taylor expansion in the Lie Series, *T*=time span of propagation, step=integration step size.
- *Step 2.* INPUT: Initialize the variables $(a, e, i, M, \omega , \Omega )$.
- *Step 3.* Integrate Hamilton’s equations of the full Hamiltonian up to time *T* to get the osculating final elements.
- *Step 4.* Compute the average of the Hamiltonian with respect to the mean anomalies of debris, Moon, Sun, and the sidereal time.
- *Step 5.* Expand up to order 3 the averaged Hamiltonian (in the extended phase space) around the reference values $e_0$, $i_0$.
- *Step 6.* Compute the generating function up to order maxSteps.
- *Step 7.* Compute the new Hamiltonian using the generating function determined at Step 6.
- *Step 8.* Compute the analytic solutions by determining the new coordinates as function of initial coordinates.
- *Step 9.* Determine the two proper elements by integrating the analytic solutions over the given interval and dividing by the length of the interval.

## Test cases: proper elements and data analysis

Let us consider a concrete case formed by, say, *N* fragments. In practical applications, our back-tracing procedure is the following: (i) we take the (initial) orbital elements of all *N* fragments at time $t=t_0$; (ii) we compute the initial proper elements from the initial orbital elements; (iii) we propagate all fragments up to a time $t=T$ to compute the (final) osculating elements; (iv) through averaging and normal form, we compute the final proper elements from the final osculating data; (v) we compare the final osculating and final proper elements with the initial orbital and initial proper elements.

Since the proper elements are quasi-integrals of motion, we expect that they retain the main features both in the initial and the final phase, thus reconnecting much better to the original elements than the propagated osculating elements. Of course, the reconnection through the proper elements is more effective in those cases in which the final osculating elements get more dispersed over time, thus losing their link with the original data. Concerning step (v), beside making a visual inspection of the plots in the planes (*a*, *i*), (*i*, *e*) of (initial and final) osculating and proper elements, we apply data analysis techniques by using the Kolmogorov-Smirnov (KS) test and the Variance Equivalence (VE) test of the errors between the osculating and proper elements taken at the initial and final times. We also compute the Pearson correlation coefficients of initial vs. final osculating elements, and initial vs. final proper elements.

Such methods, borrowed from statistical data analysis, are briefly recalled as follows^16^.

- (S1) *Kolmogorov-Smirnov test* (KST) is a goodness-of-fit test where the null hypothesis says that two datasets were taken from the same distribution, while the alternative hypothesis states that they are not taken from the same distribution. We used the predefined Mathematica$^{\copyright }$ function *KolmogorovSmirnovTest*, which returns the p-value of the statistical test. The p-value has to be compared with a significance level $\alpha$ (default is 0.05), null hypothesis being rejected for $p < \alpha$.
- (S2) *Variance Equivalence test* (VET) is a statistical tool that checks if the null hypothesis $H_0$, that the variances of two data sets are equal, is accepted or not. Depending on the datasets’ distributions and the needed assumptions, one of the following tests is applied: “Brown Forsythe”, “Conover”, “Fisher Ratio”, “Levene”. We used a Mathematica$^{\copyright }$ function called *VarianceEquivalenceTest*, that automatically chooses the most appropriate test and returns the p-value and the conclusion of the test.
- (S3) *Pearson correlation coefficient*, usually denoted by *r*, is used as a statistical measurement of the relationship between two one-dimensional datasets. It is defined as $r = \frac{Cov[X,Y]}{Var[X]Var[Y]}$ and gives a real number belonging to $[-1,1]$, where 1 means a total positive linear relationship, 0 means no relationship, and $-1$ means a total negative linear relationship between the two datasets.
- (S4) To visualize the data and to understand the main features of a distribution, one can plot the *histogram *of the dataset. This plot shows the frequency of each element from the set. This is a useful tool to compare the distributions of two or more data sets.

Among the cases available on the database “Stuff in Space” (http://stuffin.space/TLE.json), updating daily the orbit data from “Space-Track” (http://www.space-track.org/), we select the following test cases: Ariane 44lp, Atlas V Centaur, CZ-3, Titan IIIc Transtage with a number of fragments equal, respectively, to 35, 164, 139, 41. We consider the following time intervals: 25, 50, 100, 150 years.

The outcome of the data analysis is summarized in Tables 2 and 3, where we provide the comparison between different elements. Table 2 gives the results, including the p-values, about the Kolmogorov-Smirnov test and the Variance Equivalence test for the errors between osculating and proper elements at different final times. It is remarkable that both tests are always rejected, showing that the errors associated to the osculating and proper elements follow different distributions. Table 2 shows also the ratio of the root mean square errors of osculating versus proper elements, supporting that the errors associated to the osculating elements are larger than those associated to the proper elements. Table 3 gives the Pearson correlation coefficients of the initial and final, osculating and proper elements at different times.

In the supplementary material we detail the results for a fragment sample, that we take from Ariane 44lp; the supplementary material is aimed to help in reproducing the methods described in the present paper and, precisely, to compute the osculating elements at the initial and final times, to determine the normal form, to get the analytic solution and to compute the proper elements for the specific fragment. The same procedure can be implemented for the other fragments to get the results obtained in this work.

**Table 2:** The p-value of the Kolmogorov-Smirnov test and Variance Equivalence test for the errors between osculating (eoe) and proper elements (epe) at different final times (25, 50, 100, and 150 years).

Name	Time interval (yrs)	KST ecc. eoe - epe	KST inc. eoe - epe	VET ecc. eoe - epe	VET inc. eoe - epe	$\dfrac{\text {RMS ecc. eoe}}{\text {RMS ecc. epe}}$	$\dfrac{\text {RMS inc. eoe}}{\text {RMS inc. epe}}$
Ariane 44lp	25	$7.07\cdot 10^{-9}$ reject	$1.28\cdot 10^{-5}$ reject	$1.01\cdot 10^{-29}$ reject	0 reject	15.00	32.80
	50	$4.42\cdot 10^{-5}$ reject	$4.42\cdot 10^{-5}$ reject	$1.07\cdot 10^{-21}$ reject	0 reject	8.25	26.50
	100	$4.42\cdot 10^{-5}$ reject	$4.13\cdot 10^{-5}$ reject	$4.27\cdot 10^{-27}$ reject	0 reject	11.50	42.30
	150	$1.40\cdot 10^{-4}$ reject	$1.13\cdot 10^{-3}$ reject	$1.27\cdot 10^{-22}$ reject	0 reject	8.99	30.50
Atlas V Centaur	25	$7.72\cdot 10^{-16}$ reject	$2.05\cdot 10^{-15}$ reject	0 reject	0 reject	10.50	8.22
	50	$8.76\cdot 10^{-14}$ reject	$3.50\cdot 10^{-14}$ reject	0 reject	0 reject	13.70	7.91
	100	$1.38\cdot 10^{-14}$ reject	$3.54\cdot 10^{-11}$ reject	0 reject	0 reject	11.80	6.18
	150	$5.24\cdot 10^{-13}$ reject	$3.74\cdot 10^{-10}$ reject	0 reject	0 reject	10.60	7.58
CZ-3	25	$1.92\cdot 10^{-28}$ reject	$6.75\cdot 10^{-19}$ reject	0 reject	0 reject	24.50	37.30
	50	$1.85\cdot 10^{-33}$ reject	$2.01\cdot 10^{-19}$ reject	0 reject	0 reject	26.20	37.20
	100	$8.05\cdot 10^{-26}$ reject	$2.01\cdot 10^{-19}$ reject	0 reject	0 reject	26.50	37.90
	150	$8.08\cdot 10^{-30}$ reject	$5.86\cdot 10^{-20}$ reject	0 reject	0 reject	20.40	30.50
Titan IIIc Transtage	25	$6.24\cdot 10^{-4}$ reject	$9.28\cdot 10^{-6}$ reject	0 reject	0 reject	18.50	57.20
	50	$8.63\cdot 10^{-5}$ reject	$9.28\cdot 10^{-6}$ reject	0 reject	0 reject	21.40	42.00
	100	$2.92\cdot 10^{-5}$ reject	$2.92\cdot 10^{-5}$ reject	0 reject	0 reject	17.70	37.00
	150	$6.24\cdot 10^{-4}$ reject	$9.28\cdot 10^{-6}$ reject	0 reject	0 reject	17.30	36.70
Titan IIIc Transtage backward evolution	-29.5	$2.39\cdot 10^{-4}$ reject	$2.75\cdot 10^{-6}$ reject	0 reject	0 reject	30.10	59.80

The last two columns contain the ratio of the root mean square (RMS) between the osculating errors and the proper elements errors. The last row refers to an example where we back-propagate in time. A 0 p-value means a number lower than 10^−40^.

**Table 3:** The Pearson correlation coefficient obtained propagating forward in time between the initial osculating elements (ioe) and the final osculating elements (foe), between the initial osculating elements (ioe) and the final proper elements (fpe), between the initial proper elements (ipe) and the final osculating elements (foe), and between the initial proper elements (ipe) and the final proper elements (fpe) for eccentricity (ecc.) and inclination (incl.) at different final times (25, 50, 100, and 150 years).

Name	Time interval (ys)	Pearson ecc. ioe/foe	Pearson ecc. ioe/fpe	Pearson ecc. ipe/foe	Pearson ecc. ipe/fpe	Pearson incl. ioe/foe	Pearson incl. ioe/fpe	Pearson incl. ipe/foe	Pearson incl. ipe/fpe
Ariane 44lp	25	0.999368	0.999824	0.999701	0.999997	0.954275	0.976585	0.972239	0.999962
	50	0.999692	0.99982	0.999879	0.999996	0.977628	0.976828	0.983202	0.999972
	100	0.999603	0.999829	0.999735	0.999997	0.943686	0.976355	0.974763	0.99997
	150	0.999682	0.999817	0.999821	0.999995	0.957355	0.976006	0.980431	0.999951
Atlas V Centaur	25	0.989714	0.987826	0.983778	0.999885	0.982072	0.998368	0.985462	0.999769
	50	0.984756	0.989002	0.991962	0.999888	0.993723	0.998503	0.997036	0.999696
	100	0.974543	0.989358	0.985582	0.999776	0.986611	0.998284	0.988209	0.999512
	150	0.976307	0.989419	0.988603	0.999734	0.983775	0.99831	0.985861	0.999591
CZ-3	25	0.995055	0.997051	0.99769	0.99999	0.982002	0.991582	0.992269	0.999986
	50	0.995051	0.997051	0.998366	0.999991	0.98762	0.991548	0.995273	0.999991
	100	0.992798	0.997123	0.997086	0.999989	0.984668	0.991459	0.991489	0.999989
	150	0.994158	0.997079	0.997453	0.999983	0.984414	0.991584	0.991767	0.999982
Titan IIIc Transtage	25	0.998889	0.999202	0.999495	0.999997	0.889829	0.95929	0.941887	0.999969
	50	0.998572	0.999209	0.999455	0.999997	0.906092	0.95893	0.960931	0.999944
	100	0.99899	0.9992	0.99955	0.999997	0.916836	0.959268	0.959239	0.999932
	150	0.998927	0.999199	0.9996	0.999997	0.873322	0.959531	0.934404	0.999904
Titan IIIc Transtage backward evolution	− 29.5	0.99886	0.999188	0.999641	0.999997	0.888539	0.959189	0.954393	0.999947

The last row refers to an example where we back-propagate in time.

**Figure 2:**
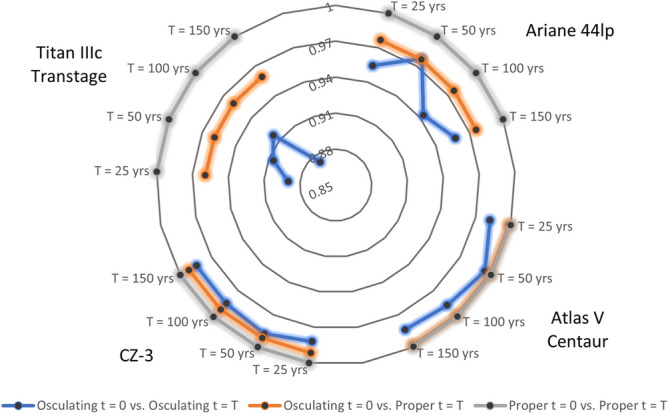
Comparison of the Pearson correlation coefficient for our test cases in three different combinations of the data concerning the inclination: initial osculating inclination vs final osculating inclination (blue line), initial osculating inclination vs final proper inclination (orange line), and initial proper inclination vs final proper inclination (gray line). As final times we take 25, 50, 100, 150 years.

Using the data in Table 3, Fig. 2 summarizes the Pearson correlation coefficient between the initial data and the final osculating and proper inclination, as well as the initial and final proper inclination. In all sample cases, the correlation between the initial and final proper elements is always close to 1, while using the other sets we obtain discrepancies between the correlations of the initial and final states.

In the case of Titan IIIc Transtage there is a weak correlation between initial and final osculating elements, a better Pearson coefficient between initial osculating elements and final proper elements, and an almost perfect fit of initial and final proper elements. The other three sample cases have a similar behavior.

### Atlas V Centaur

As an example, we analyze in detail the statistics of Atlas V Centaur and we describe the numbers shown in Tables 2 and 3. Table 2 shows that the KS test and the VE test are always rejected, both for eccentricity and inclination, at all times we investigated, namely 25, 50, 100, 150 years. Hence, the errors for osculating and proper elements follow different distributions with the errors associated to the osculating elements being larger than those of the proper elements.

The Pearson correlation coefficient in Table 3 tends to be constant when we compare the proper elements at different times. This result confirms the near constancy of the proper elements for a long period of time.

**Figure 3:**
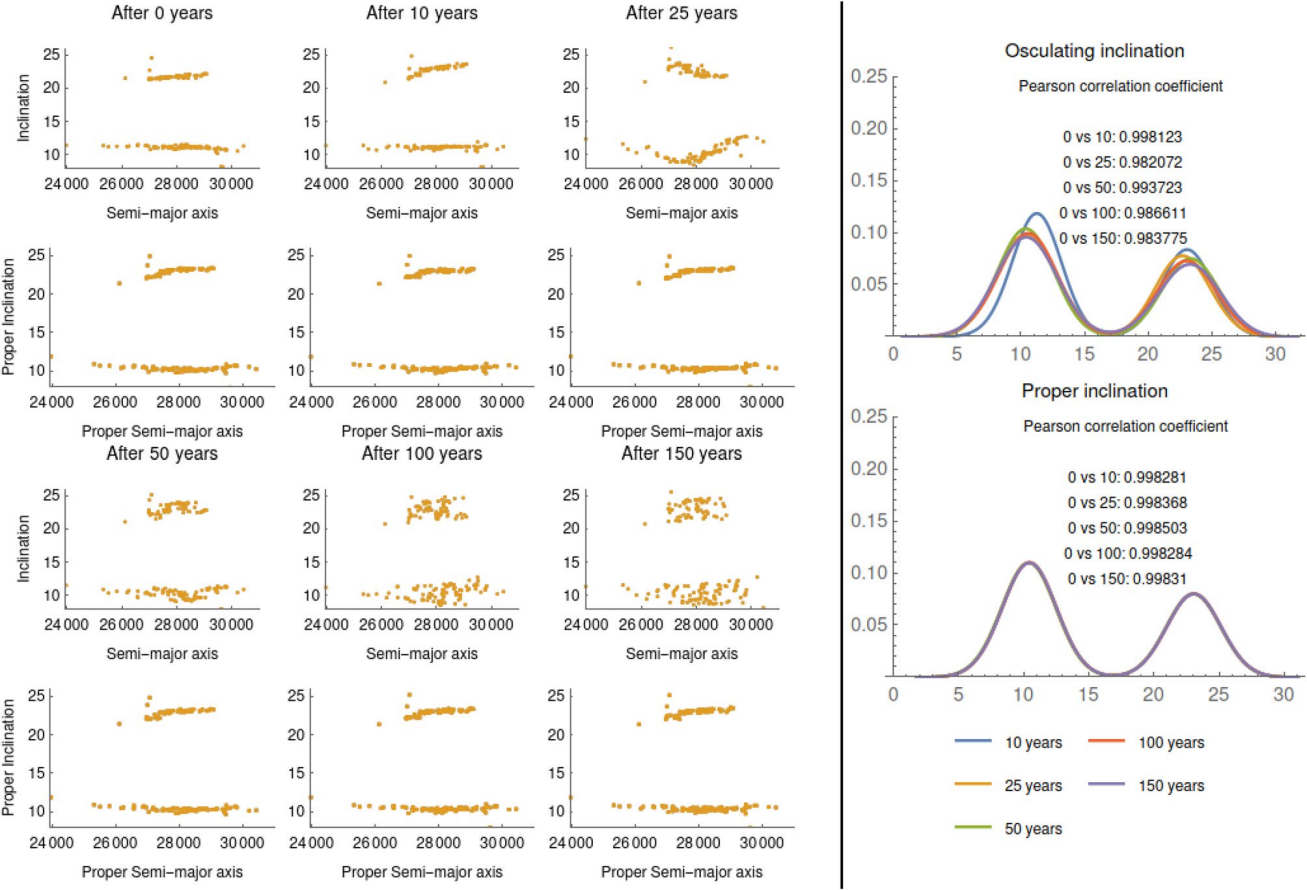
Case of Atlas V Centaur. Comparison between the evolution of the osculating *a*-*i* and the evolution of the proper *a*-*i* (left). Histogram of the osculating inclination (top-right), and the proper inclination (bottom-right).

Figure 3 shows the evolution of the osculating elements in the plane *a*-*i* compared with the evolution of the proper elements in the same plane (left); it also shows the distribution of the inclination (right) for the times 10, 25, 50, 100, 150 years in case of osculating (top) and proper (bottom) elements.

As it can be seen from the plots in Fig. 3, the osculating inclination starts to spread around 25 years; the spread increases with time. On the contrary, the proper inclination is kept almost constant, thus allowing to reconstruct the distribution at the initial time. This fact is also confirmed by the histograms and the associated Pearson correlation coefficients.

### Titan IIIc Transtage

It is known that^17^ on February 21, 1992 an explosion of Titan IIIc Transtage produced several debris. All debris have been tracked and their coordinates at the present time can be found on “Space track”. We test our procedure, assuming to ignore the break-up time and propagating backward all fragments for a period of time equal to 29.5 years. The following results confirm the validity of the procedure based on the computation and comparison of the proper elements. In fact, like in the other cases, the KS and VE tests are rejected for all times with errors bigger for the osculating than for the proper elements. Beside, comparing the osculating elements at the present time and at the final time we obtain a Pearson correlation coefficient equal to 0.99886 for the eccentricity and 0.888539 for the inclination. On the other hand, comparing the proper elements at present and backward in time, we find a Pearson correlation coefficient equal to 0.999997 for the eccentricity and 0.999947 for the inclination.

### Two mixed cases

**Figure 4:**
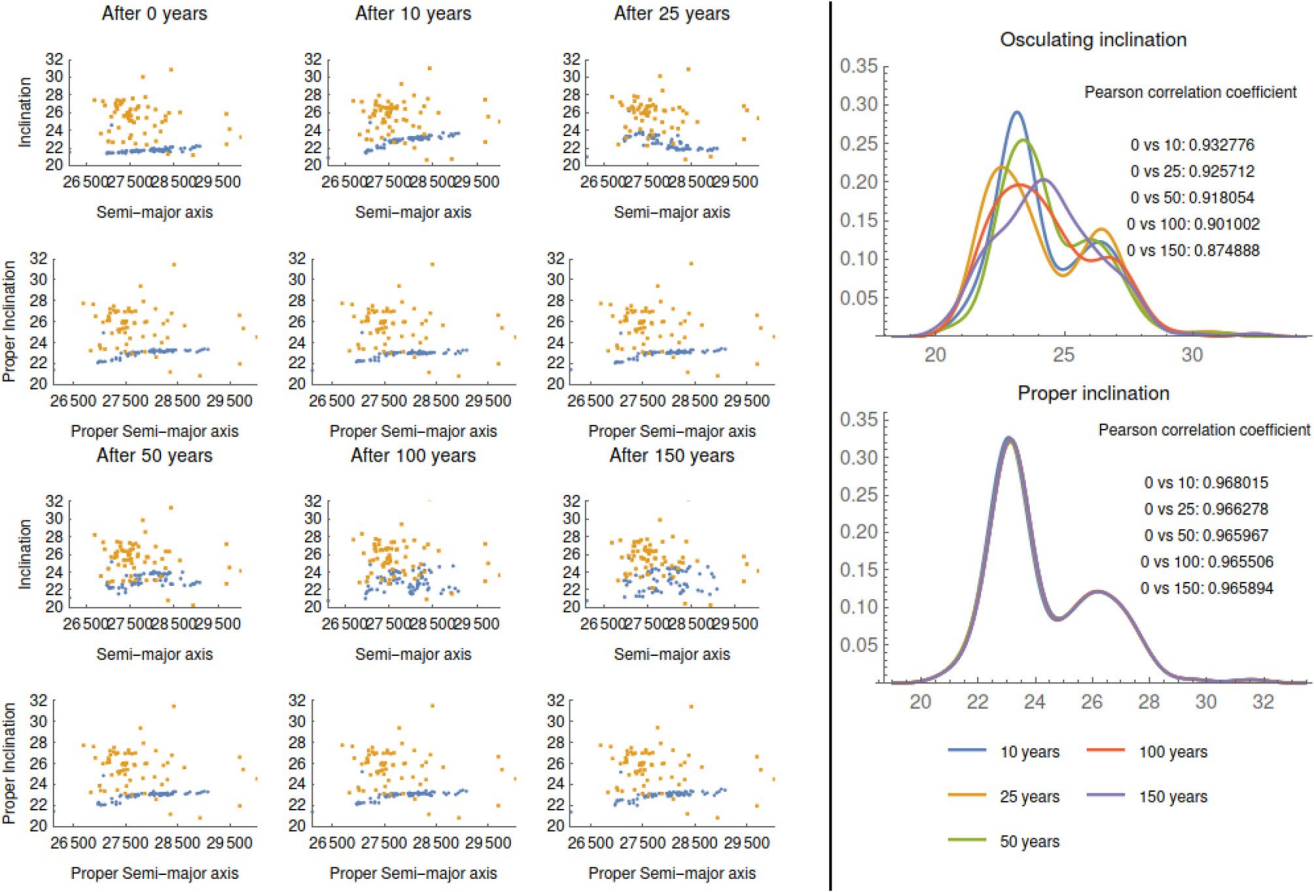
Mixed case between CZ-3 and Atlas V Centaur. Evolution of both the osculating and proper inclination over 10, 25, 50, 100, 150 years (left); the histograms and the Pearson correlation coefficients between osculating and proper inclinations (right).

We finally test our method by mixing the cases of CZ-3 and Atlas V Centaur; the results are given in Fig. 4, which shows the evolution of the osculating and proper inclinations at different times (10, 25, 50, 100, 150 years). Through the proper elements, we succeed to distinguish two different clouds. In fact, while the osculating elements change so much that we cannot recognize the two groups after 50 years, the proper elements keep constant over all periods of time. This conclusion is supported by the comparison of the Pearson correlation coefficients and the histograms as in the right side of Fig. 4.

## Conclusions and perspectives

The potentiality of the procedure of computing hierarchical proper elements for the space debris is witnessed by the four sample cases that we have analyzed in the previous sections and whose results are summarized in Tables 2, 3 and Fig. 2. Based on a solid mathematical method, our approach provides a reliable and effective way to connect the proper elements of a set of fragments to a specific break-up event. Even not knowing the break-up time, we can propagate backward the elements of the space debris to reconnect the debris to the parent body; we show the effectiveness of this procedure in the specific example of Titan IIIc Transtage. We have also shown a sample where the procedure allows one to distinguish between fragments generated by nearby break-up events. We stress that the computation of the proper elements can be extended to encompass limit cases of very small/large eccentricities and inclinations, as well as in the case of orbital elements close to resonances^18,19^. Such cases can be dealt with a dedicated perturbation theory that generalizes our approach.

## Supplementary Information

Supplementary Information.
