# Supplementary material for: Reconnecting groups of space debris to their parent body through proper elements
Source: Sci Rep. 2021 Nov 22;11:22676. doi: 10.1038/s41598-021-02010-x (PMC8608945; doi:10.1038/s41598-021-02010-x)
Supplement: Supplementary file 1 — Supplementary Information. [file 41598_2021_2010_MOESM1_ESM.pdf]

Reconnecting groups of space debris to their parent body through proper elements: Supplementary Material

Alessandra Celletti[1,*,+], **Giuseppe Pucacco**[2,+], **and Tudor Vartolomei**[1,+]

[1]Department of Mathematics, University of Rome Tor Vergata; Via della Ricerca Scientifica 1, 00133 Rome, Italy
[2]Department of Physics, University of Rome Tor Vergata; Via della Ricerca Scientifica 1, 00133 Rome, Italy
[*]celletti@mat.uniroma2.it
[+]these authors contributed equally to this work

ABSTRACT

This text contains supplementary material for the paper "Reconnecting groups of space debris to their parent body through proper elements".

Materials and method

This document contains: the explicit expressions in orbital elements of the Hamiltonians of Moon and Sun, the non-averaged geopotential including the terms J_2 and J_3, and some parts of the algorithm to compute the proper elements, the orbital data for one sample case, namely Ariane 44 lp, the explicit computations of the normal form and the proper elements for two fragments of Ariane 44 lp and some details on the statistical data analysis.

Hamiltonians of Moon and Sun.

The expressions of the Hamiltonians for Moon and Sun are obtained by implementing the formulae in (*21*).

We report below the contribution to the Hamiltonian due to the Moon, expanded to second order in orbital elements (compare with (*2*), (*9*), (*10*), (*11*)).

$$
\begin{aligned}
& H_M = -3.06238\cdot 10^{-6}a^2 - 4.59357\cdot 10^{-6}a^2e^2 - 5.95633\cdot 10^{-7}a^2e^2\cos(2\omega-2\Omega)\\
+\ & 1.19127\cdot 10^{-6}a^2e^2\cos(i)\cos(2\omega-2\Omega) - 5.95633\cdot 10^{-7}a^2e^2\cos(i)^2\cos(2\omega-2\Omega)\\
-\ & 4.76507\cdot 10^{-7}a^2\cos(2\Omega) - 7.1476\cdot 10^{-7}a^2e^2\cos(2\Omega)\\
+\ & 4.76507\cdot 10^{-7}a^2\cos(i)^2\cos(2\Omega) + 7.1476\cdot 10^{-7}a^2e^2\cos(i)^2\cos(2\Omega)\\
-\ & 5.95633\cdot 10^{-7}a^2e^2\cos(2\omega+2\Omega) - 1.19127\cdot 10^{-6}a^2e^2\cos(i)\cos(2\omega+2\Omega)\\
-\ & 5.95633\cdot 10^{-7}a^2e^2\cos(i)^2\cos(2\omega+2\Omega) - 5.43353\cdot 10^{-11}a^2e^2\cos(2\omega-2\Omega-2\Omega_M)\\
+\ & 1.08671\cdot 10^{-10}a^2e^2\cos(i)\cos(2\omega-2\Omega-2\Omega_M) - 5.43353\cdot 10^{-11}a^2e^2\cos(i)^2\cos(2\omega-2\Omega-2\Omega_M)\\
-\ & 2.34714\cdot 10^{-8}a^2\cos(2\Omega-2\Omega_M) - 3.5207\cdot 10^{-8}a^2e^2\cos(2\Omega-2\Omega_M)\\
+\ & 2.34714\cdot 10^{-8}a^2\cos(i)^2\cos(2\Omega-2\Omega_M) + 3.5207\cdot 10^{-8}a^2e^2\cos(i)^2\cos(2\Omega-2\Omega_M)\\
-\ & 2.93392\cdot 10^{-8}a^2e^2\cos(2\omega+2\Omega-2\Omega_M) - 5.86784\cdot 10^{-8}a^2e^2\cos(i)\cos(2\omega+2\Omega-2\Omega_M)\\
-\ & 2.93392\cdot 10^{-8}a^2e^2\cos(i)^2\cos(2\omega+2\Omega-2\Omega_M) + 1.1402\cdot 10^{-8}a^2e^2\cos(2\omega-2\Omega-\Omega_M)\\
-\ & 2.28039\cdot 10^{-8}a^2e^2\cos(i)\cos(2\omega-2\Omega-\Omega_M) + 1.1402\cdot 10^{-8}a^2e^2\cos(i)^2\cos(2\omega-2\Omega-\Omega_M)\\
-\ & 2.11959\cdot 10^{-7}a^2\cos(2\Omega-\Omega_M) - 3.17939\cdot 10^{-7}a^2e^2\cos(2\Omega-\Omega_M)\\
+\ & 2.11959\cdot 10^{-7}a^2\cos(i)^2\cos(2\Omega-\Omega_M) + 3.17939\cdot 10^{-7}a^2e^2\cos(i)^2\cos(2\Omega-\Omega_M)\\
-\ & 2.64949\cdot 10^{-7}a^2e^2\cos(2\omega+2\Omega-\Omega_M) - 5.29898\cdot 10^{-7}a^2e^2\cos(i)\cos(2\omega+2\Omega-\Omega_M)\\
-\ & 2.64949\cdot 10^{-7}a^2e^2\cos(i)^2\cos(2\omega+2\Omega-\Omega_M) + 4.05675\cdot 10^{-7}a^2\cos(\Omega_M)\\
+\ & 6.08513\cdot 10^{-7}a^2e^2\cos(\Omega_M) - 4.04032\cdot 10^{-9}a^2\cos(2\Omega_M) - 6.06047\cdot 10^{-9}a^2e^2\cos(2\Omega_M)\\
-\ & 2.64949\cdot 10^{-7}a^2e^2\cos(2\omega-2\Omega+\Omega_M) + 5.29898\cdot 10^{-7}a^2e^2\cos(i)\cos(2\omega-2\Omega+\Omega_M)
\end{aligned}
$$

$$
\begin{aligned}
&- \quad 2.64949\cdot 10^{-7}a^2e^2\cos(i)^2\cos(2\omega-2\Omega+\Omega_M)+9.12157\cdot 10^{-9}a^2\cos(2\Omega+\Omega_M)\\
&+ \quad 1.36823\cdot 10^{-8}a^2e^2\cos(2\Omega+\Omega_M)-9.12157\cdot 10^{-9}a^2\cos(i)^2\cos(2\Omega+\Omega_M)\\
&- \quad 1.36823\cdot 10^{-8}a^2e^2\cos(i)^2\cos(2\Omega+\Omega_M)+1.1402\cdot 10^{-8}a^2e^2\cos(2\omega+2\Omega+\Omega_M)\\
&+ \quad 2.28039\cdot 10^{-8}a^2e^2\cos(i)\cos(2\omega+2\Omega+\Omega_M)+1.1402\cdot 10^{-8}a^2e^2\cos(i)^2\cos(2\omega+2\Omega+\Omega_M)\\
&- \quad 2.93392\cdot 10^{-8}a^2e^2\cos(2\omega-2\Omega+2\Omega_M)+5.86784\cdot 10^{-8}a^2e^2\cos(i)\cos(2\omega-2\Omega+2\Omega_M)\\
&- \quad 2.93392\cdot 10^{-8}a^2e^2\cos(i)^2\cos(2\omega-2\Omega+2\Omega_M)-4.34683\cdot 10^{-11}a^2\cos(2\Omega+2\Omega_M)\\
&- \quad 6.52024\cdot 10^{-11}a^2e^2\cos(2\Omega+2\Omega_M)+4.34683\cdot 10^{-11}a^2\cos(i)^2\cos(2\Omega+2\Omega_M)\\
&+ \quad 6.52024\cdot 10^{-11}a^2e^2\cos(i)^2\cos(2\Omega+2\Omega_M)-5.43353\cdot 10^{-11}a^2e^2\cos(2\omega+2\Omega+2\Omega_M)\\
&- \quad 1.08671\cdot 10^{-10}a^2e^2\cos(i)\cos(2\omega+2\Omega+2\Omega_M)-5.43353\cdot 10^{-11}a^2e^2\cos(i)^2\cos(2\omega+2\Omega+2\Omega_M)\\
&- \quad 5.49537\cdot 10^{-6}a^2e^2\cos(2\omega-\Omega)\sin(i)+5.49537\cdot 10^{-6}a^2e^2\cos(i)\cos(2\omega-\Omega)\sin(i)\\
&- \quad 4.39629\cdot 10^{-6}a^2\cos(i)\cos(\Omega)\sin(i)-6.59444\cdot 10^{-6}a^2e^2\cos(i)\cos(\Omega)\sin(i)\\
&+ \quad 5.49537\cdot 10^{-6}a^2e^2\cos(2\omega+\Omega)\sin(i)+5.49537\cdot 10^{-6}a^2e^2\cos(i)\cos(2\omega+\Omega)\sin(i)\\
&- \quad 1.04769\cdot 10^{-9}a^2e^2\cos(2\omega-\Omega-2\Omega_M)\sin(i)+1.04769\cdot 10^{-9}a^2e^2\cos(i)\cos(2\omega-\Omega-2\Omega_M)\sin(i)\\
&+ \quad 1.94763\cdot 10^{-8}a^2\cos(i)\cos(\Omega-2\Omega_M)\sin(i)+2.92145\cdot 10^{-8}a^2e^2\cos(i)\cos(\Omega-2\Omega_M)\sin(i)\\
&- \quad 2.43454\cdot 10^{-8}a^2e^2\cos(2\omega+\Omega-2\Omega_M)\sin(i)-2.43454\cdot 10^{-8}a^2e^2\cos(i)\cos(2\omega+\Omega-2\Omega_M)\sin(i)\\
&+ \quad 1.62524\cdot 10^{-7}a^2e^2\cos(2\omega-\Omega-\Omega_M)\sin(i)-1.62524\cdot 10^{-7}a^2e^2\cos(i)\cos(2\omega-\Omega-\Omega_M)\sin(i)\\
&- \quad 8.89837\cdot 10^{-7}a^2\cos(i)\cos(\Omega-\Omega_M)\sin(i)-1.33475\cdot 10^{-6}a^2e^2\cos(i)\cos(\Omega-\Omega_M)\sin(i)\\
&+ \quad 1.1123\cdot 10^{-6}a^2e^2\cos(2\omega+\Omega-\Omega_M)\sin(i)+1.1123\cdot 10^{-6}a^2e^2\cos(i)\cos(2\omega+\Omega-\Omega_M)\sin(i)\\
&- \quad 1.1123\cdot 10^{-6}a^2e^2\cos(2\omega-\Omega+\Omega_M)\sin(i)+1.1123\cdot 10^{-6}a^2e^2\cos(i)\cos(2\omega-\Omega+\Omega_M)\sin(i)\\
&+ \quad 1.30019\cdot 10^{-7}a^2\cos(i)\cos(\Omega+\Omega_M)\sin(i)+1.95029\cdot 10^{-7}a^2e^2\cos(i)\cos(\Omega+\Omega_M)\sin(i)\\
&- \quad 1.62524\cdot 10^{-7}a^2e^2\cos(2\omega+\Omega+\Omega_M)\sin(i)-1.62524\cdot 10^{-7}a^2e^2\cos(i)\cos(2\omega+\Omega+\Omega_M)\sin(i)\\
&+ \quad 2.43454\cdot 10^{-8}a^2e^2\cos(2\omega-\Omega+2\Omega_M)\sin(i)-2.43454\cdot 10^{-8}a^2e^2\cos(i)\cos(2\omega-\Omega+2\Omega_M)\sin(i)\\
&- \quad 8.38154\cdot 10^{-10}a^2\cos(i)\cos(\Omega+2\Omega_M)\sin(i)-1.25723\cdot 10^{-9}a^2e^2\cos(i)\cos(\Omega+2\Omega_M)\sin(i)\\
&+ \quad 1.04769\cdot 10^{-9}a^2e^2\cos(2\omega+\Omega+2\Omega_M)\sin(i)+1.04769\cdot 10^{-9}a^2e^2\cos(i)\cos(2\omega+\Omega+2\Omega_M)\sin(i)\\
&+ \quad 4.59357\cdot 10^{-6}a^2\sin(i)^2+6.89035\cdot 10^{-6}a^2e^2\sin(i)^2-0.0000114839a^2e^2\cos(2\omega)\sin(i)^2\\
&- \quad 7.57559\cdot 10^{-9}a^2e^2\cos(2\omega-2\Omega_M)\sin(i)^2+7.60641\cdot 10^{-7}a^2e^2\cos(2\omega-\Omega_M)\sin(i)^2\\
&- \quad 6.08513\cdot 10^{-7}a^2\cos(\Omega_M)\sin(i)^2-9.12769\cdot 10^{-7}a^2e^2\cos(\Omega_M)\sin(i)^2\\
&+ \quad 6.06047\cdot 10^{-9}a^2\cos(2\Omega_M)\sin(i)^2+9.09071\cdot 10^{-9}a^2e^2\cos(2\Omega_M)\sin(i)^2\\
&+ \quad 7.60641\cdot 10^{-7}a^2e^2\cos(2\omega+\Omega_M)\sin(i)^2-7.57559\cdot 10^{-9}a^2e^2\cos(2\omega+2\Omega_M)\sin(i)^2
\end{aligned}
$$

Similarly to the Moon, we report below the contribution to the Hamiltonian due to the Sun, expanded to second order in orbital elements.

$$
\begin{aligned}
& \quad H_S=-1.42243\cdot 10^{-6}a^2-2.13364\cdot 10^{-6}a^2e^2-2.76662\cdot 10^{-7}a^2e^2\cos(2\omega-2\Omega)\\
&+ \quad 5.53324\cdot 10^{-7}a^2e^2\cos(i)\cos(2\omega-2\Omega)-2.76662\cdot 10^{-7}a^2e^2\cos(i)^2\cos(2\omega-2\Omega)\\
&- \quad 2.2133\cdot 10^{-7}a^2\cos(2\Omega)-3.31995\cdot 10^{-7}a^2e^2\cos(2\Omega)+2.2133\cdot 10^{-7}a^2\cos(i)^2\cos(2\Omega)\\
&+ \quad 3.31995\cdot 10^{-7}a^2e^2\cos(i)^2\cos(2\Omega)-2.76662\cdot 10^{-7}a^2e^2\cos(2\omega+2\Omega)\\
&- \quad 5.53324\cdot 10^{-7}a^2e^2\cos(i)\cos(2\omega+2\Omega)-2.76662\cdot 10^{-7}a^2e^2\cos(i)^2\cos(2\omega+2\Omega)\\
&- \quad 2.55251\cdot 10^{-6}a^2e^2\cos(2\omega-\Omega)\sin(i)+2.55251\cdot 10^{-6}a^2e^2\cos(i)\cos(2\omega-\Omega)\sin(i)\\
&- \quad 2.04201\cdot 10^{-6}a^2\cos(i)\cos(\Omega)\sin(i)-3.06301\cdot 10^{-6}a^2e^2\cos(i)\cos(\Omega)\sin(i)\\
&+ \quad 2.55251\cdot 10^{-6}a^2e^2\cos(2\omega+\Omega)\sin(i)+2.55251\cdot 10^{-6}a^2e^2\cos(i)\cos(2\omega+\Omega)\sin(i)\\
&+ \quad 2.13364\cdot 10^{-6}a^2\sin(i)^2+3.20046\cdot 10^{-6}a^2e^2\sin(i)^2-5.33411\cdot 10^{-6}a^2e^2\cos(2\omega)\sin(i)^2\ .
\end{aligned}
$$

For later reference we introduce the following terminology concerning the angle variables describing the space debris problem:

- *fast* variables are M, θ with periods of the order of hours or days;
- *semi-fast* variables are M_M, M_S with periods of the order of months, years;

- *slow* variables are ω, Ω, ω_M, Ω_M, ω_S, Ω_S with periods of years.

Non-averaged geopotential including the terms J_2 and J_3.

We report below the expression of the geopotential expanded in spherical harmonics and including the terms associated to the coefficients J_2 and J_3. This Hamiltonian, together with the averaged Hamiltonians of Moon and Sun, allows us to compute the osculating elements after a fixed time interval by integrating Hamilton's equations from given initial conditions.

$$
\begin{aligned}
V_{J_2J_3} = {} & \frac{1}{a^4}((3.08969\cdot 10^{-6}a)/(\sqrt{1-e^2}(-1+e^2))+(9.26907\cdot 10^{-6}a\cos(2i))/(\sqrt{1-e^2}(-1+e^2)) \\
+ \; & (-0.0000370763+0.0000556144\sin^2(i))ae\cos(M)-0.0000185381\sin^2(i)a\cos(2(M+\omega)) \\
+ \; & 0.0000463453\sin^2(i)ae^2\cos(2(M+\omega))+9.26907\cdot 10^{-6}\sin^2(i)ae\cos(M+2\omega) \\
- \; & 0.0000648835\sin^2(i)ae\cos(3M+2\omega)-1.1344\cdot 10^{-7}\sin^2(i)e^2\cos(3.26323-5M-3\omega-\Omega+\theta) \\
- \; & 1.1344\cdot 10^{-7}\cos(i)\sin^2(i)e^2\cos(3.26323-5M-3\omega-\Omega+\theta)-3.57291\cdot 10^{-8}\sin^2(i)e\cos(3.26323-4M-3\omega-\Omega+\theta) \\
- \; & 3.57291\cdot 10^{-8}\cos(i)\sin^2(i)e\cos(3.26323-4M-3\omega-\Omega+\theta)-7.14581\cdot 10^{-9}\sin^2(i)\cos(3.26323-3M-3\omega-\Omega+\theta) \\
- \; & 7.14581\cdot 10^{-9}\cos(i)\sin^2(i)\cos(3.26323-3M-3\omega-\Omega+\theta)+4.28749\cdot 10^{-8}\sin^2(i)e^2\cos(3.26323-3M-3\omega-\Omega+\theta) \\
+ \; & 4.28749\cdot 10^{-8}\cos(i)\sin^2(i)e^2\cos(3.26323-3M-3\omega-\Omega+\theta)-3.78728\cdot 10^{-8}e^2\cos(3.26323-3M-\omega-\Omega+\theta) \\
- \; & 3.78728\cdot 10^{-8}\cos(i)e^2\cos(3.26323-3M-\omega-\Omega+\theta)+4.7341\cdot 10^{-8}\sin^2(i)e^2\cos(3.26323-3M-\omega-\Omega+\theta) \\
+ \; & 1.42023\cdot 10^{-7}\cos(i)\sin^2(i)e^2\cos(3.26323-3M-\omega-\Omega+\theta)-1.71499\cdot 10^{-8}e\cos(3.26323-2M-\omega-\Omega+\theta) \\
- \; & 1.71499\cdot 10^{-8}\cos(i)e\cos(3.26323-2M-\omega-\Omega+\theta)+2.14374\cdot 10^{-8}\sin^2(i)e\cos(3.26323-2M-\omega-\Omega+\theta) \\
+ \; & 6.43123\cdot 10^{-8}\cos(i)\sin^2(i)e\cos(3.26323-2M-\omega-\Omega+\theta)-5.71665\cdot 10^{-9}\cos(3.26323-1M-\omega-\Omega+\theta) \\
- \; & 5.71665\cdot 10^{-9}\cos(i)\cos(3.26323-M-\omega-\Omega+\theta)+7.14581\cdot 10^{-9}\sin^2(i)\cos(3.26323-M-\omega-\Omega+\theta) \\
+ \; & 2.14374\cdot 10^{-8}\cos(i)\sin^2(i)\cos(3.26323-M-\omega-\Omega+\theta)-1.14333\cdot 10^{-8}e^2\cos(3.26323-M-\omega-\Omega+\theta) \\
- \; & 1.14333\cdot 10^{-8}\cos(i)e^2\cos(3.26323-M-\omega-\Omega+\theta)+1.42916\cdot 10^{-8}\sin^2(i)e^2\cos(3.26323-M-\omega-\Omega+\theta) \\
+ \; & 4.28749\cdot 10^{-8}\cos(i)\sin^2(i)e^2\cos(3.26323-M-\omega-\Omega+\theta)-7.86039\cdot 10^{-9}e^2\cos(3.26323+M-\omega-\Omega+\theta) \\
- \; & 7.86039\cdot 10^{-9}\cos(i)e^2\cos(3.26323+M-\omega-\Omega+\theta)+9.82549\cdot 10^{-9}\sin^2(i)e^2\cos(3.26323+M-\omega-\Omega+\theta) \\
+ \; & 2.94765\cdot 10^{-8}\cos(i)\sin^2(i)e^2\cos(3.26323+M-\omega-\Omega+\theta)-7.86039\cdot 10^{-9}e^2\cos(3.26323-M+\omega-\Omega+\theta) \\
+ \; & 7.86039\cdot 10^{-9}\cos(i)e^2\cos(3.26323-M+\omega-\Omega+\theta)+9.82549\cdot 10^{-9}\sin^2(i)e^2\cos(3.26323-M+\omega-\Omega+\theta) \\
- \; & 2.94765\cdot 10^{-8}\cos(i)\sin^2(i)e^2\cos(3.26323-M+\omega-\Omega+\theta)-5.71665\cdot 10^{-9}\cos(3.26323+M+\omega-\Omega+\theta) \\
+ \; & 5.71665\cdot 10^{-9}\cos(i)\cos(3.26323+M+\omega-\Omega+\theta)+7.14581\cdot 10^{-9}\sin^2(i)\cos(3.26323+M+\omega-\Omega+\theta) \\
- \; & 2.14374\cdot 10^{-8}\cos(i)\sin^2(i)\cos(3.26323+M+\omega-\Omega+\theta)-1.14333\cdot 10^{-8}e^2\cos(3.26323+M+\omega-\Omega+\theta) \\
+ \; & 1.14333\cdot 10^{-8}\cos(i)e^2\cos(3.26323+M+\omega-\Omega+\theta)+1.42916\cdot 10^{-8}\sin^2(i)e^2\cos(3.26323+M+\omega-\Omega+\theta) \\
- \; & 4.28749\cdot 10^{-8}\cos(i)\sin^2(i)e^2\cos(3.26323+M+\omega-\Omega+\theta)-1.71499\cdot 10^{-8}e\cos(3.26323+2M+\omega-\Omega+\theta) \\
+ \; & 1.71499\cdot 10^{-8}\cos(i)e\cos(3.26323+2M+\omega-\Omega+\theta)+2.14374\cdot 10^{-8}\sin^2(i)e\cos(3.26323+2M+\omega-\Omega+\theta) \\
- \; & 6.43123\cdot 10^{-8}\cos(i)\sin^2(i)e\cos(3.26323+2M+\omega-\Omega+\theta)-3.78728\cdot 10^{-8}e^2\cos(3.26323+3M+\omega-\Omega+\theta) \\
+ \; & 3.78728\cdot 10^{-8}\cos(i)e^2\cos(3.26323+3M+\omega-\Omega+\theta)+4.7341\cdot 10^{-8}\sin^2(i)e^2\cos(3.26323+3M+\omega-\Omega+\theta) \\
- \; & 1.42023\cdot 10^{-7}\cos(i)\sin^2(i)e^2\cos(3.26323+3M+\omega-\Omega+\theta)-3.57291\cdot 10^{-8}\sin^2(i)e\cos(3.26323+4M+3\omega-\Omega+\theta) \\
+ \; & 3.57291\cdot 10^{-8}\cos(i)\sin^2(i)e\cos(3.26323+4M+3\omega-\Omega+\theta)-1.1344\cdot 10^{-7}\sin^2(i)e^2\cos(3.26323+5M+3\omega-\Omega+\theta) \\
+ \; & 1.1344\cdot 10^{-7}\cos(i)\sin^2(i)e^2\cos(3.26323+5M+3\omega-\Omega+\theta) \\
- \; & (6.21781\cdot 10^{-8}\sin^2(i)a\cos(2.62049-2\Omega+2\theta))/(\sqrt{1-e^2}(-1+e^2)) \\
+ \; & 9.32671\cdot 10^{-8}ae\cos(2.62049-M-2\Omega+2\theta)-9.32671\cdot 10^{-8}\cos^2(i)ae\cos(2.62049-M-2\Omega+2\theta) \\
+ \; & 9.32671\cdot 10^{-8}ae\cos(2.62049+M-2\Omega+2\theta)-9.32671\cdot 10^{-8}\cos^2(i)ae\cos(2.62049+M-2\Omega+2\theta) \\
+ \; & 1.08812\cdot 10^{-7}ae\cos(2.62049-3M-2\omega-2\Omega+2\theta)+2.17623\cdot 10^{-7}\cos(i)ae\cos(2.62049-3M-2\omega-2\Omega+2\theta) \\
+ \; & 1.08812\cdot 10^{-7}\cos^2(i)ae\cos(2.62049-3M-2\omega-2\Omega+2\theta)+3.1089\cdot 10^{-8}a\cos(2.62049-2M-2\omega-2\Omega+2\theta) \\
+ \; & 6.21781\cdot 10^{-8}\cos(i)a\cos(2.62049-2M-2\omega-2\Omega+2\theta)+3.1089\cdot 10^{-8}\cos^2(i)a\cos(2.62049-2M-2\omega-2\Omega+2\theta) \\
- \; & 7.77226\cdot 10^{-8}ae^2\cos(2.62049-2M-2\omega-2\Omega+2\theta)-1.55445\cdot 10^{-7}\cos(i)ae^2\cos(2.62049-2M-2\omega-2\Omega+2\theta) \\
- \; & 7.77226\cdot 10^{-8}\cos^2(i)ae^2\cos(2.62049-2M-2\omega-2\Omega+2\theta)-1.55445\cdot 10^{-8}ae\cos(2.62049-M-2\omega-2\Omega+2\theta) \\
- \; & 3.1089\cdot 10^{-8}\cos(i)ae\cos(2.62049-M-2\omega-2\Omega+2\theta)-1.55445\cdot 10^{-8}\cos^2(i)ae\cos(2.62049-M-2\omega-2\Omega+2\theta)
\end{aligned}
$$

$$
\begin{aligned}
- & \; 1.55445 \cdot 10^{-8} ae\cos(2.62049+M+2\omega-2\Omega+2\theta)+3.1089\cdot 10^{-8}\cos(i)ae\cos(2.62049+M+2\omega-2\Omega+2\theta) \\
- & \; 1.55445 \cdot 10^{-8}\cos^2(i)ae\cos(2.62049+M+2\omega-2\Omega+2\theta)+3.1089\cdot 10^{-8}a\cos(2.62049+2M+2\omega-2\Omega+2\theta) \\
- & \; 6.21781 \cdot 10^{-8}\cos(i)a\cos(2.62049+2M+2\omega-2\Omega+2\theta)+3.1089\cdot 10^{-8}\cos^2(i)a\cos(2.62049+2M+2\omega-2\Omega+2\theta) \\
- & \; 7.77226 \cdot 10^{-8}ae^2\cos(2.62049+2M+2\omega-2\Omega+2\theta)+1.55445\cdot 10^{-7}\cos(i)ae^2\cos(2.62049+2M+2\omega-2\Omega+2\theta) \\
- & \; 7.77226 \cdot 10^{-8}\cos^2(i)ae^2\cos(2.62049+2M+2\omega-2\Omega+2\theta)+1.08812\cdot 10^{-7}ae\cos(2.62049+3M+2\omega-2\Omega+2\theta) \\
- & \; 2.17623 \cdot 10^{-7}\cos(i)ae\cos(2.62049+3M+2\omega-2\Omega+2\theta)+1.08812\cdot 10^{-7}\cos^2(i)ae\cos(2.62049+3M+2\omega-2\Omega+2\theta) \\
+ & \; 2.27334 \cdot 10^{-8}e^2\cos(4.24077-5M-3\omega-3\Omega+3\theta)+6.82003\cdot 10^{-8}\cos(i)e^2\cos(4.24077-5M-3\omega-3\Omega+3\theta) \\
+ & \; 6.82003 \cdot 10^{-8}\cos^2(i)e^2\cos(4.24077-5M-3\omega-3\Omega+3\theta)+2.27334\cdot 10^{-8}\cos^3(i)e^2\cos(4.24077-5M-3\omega-3\Omega+3\theta) \\
+ & \; 2.84616 \cdot 10^{-8}e^2\cos(4.24077-3M-\omega-3\Omega+3\theta)+2.84616\cdot 10^{-8}\cos(i)e^2\cos(4.24077-3M-\omega-3\Omega+3\theta) \\
- & \; 2.84616 \cdot 10^{-8}\cos^2(i)e^2\cos(4.24077-3M-\omega-3\Omega+3\theta)-2.84616\cdot 10^{-8}\cos^3(i)e^2\cos(4.24077-3M-\omega-3\Omega+3\theta) \\
+ & \; 1.28882 \cdot 10^{-8}e\cos(4.24077-2M-\omega-3\Omega+3\theta)+1.28882\cdot 10^{-8}\cos(i)e\cos(4.24077-2M-\omega-3\Omega+3\theta) \\
- & \; 1.28882 \cdot 10^{-8}\cos^2(i)e\cos(4.24077-2M-\omega-3\Omega+3\theta)-1.28882\cdot 10^{-8}\cos^3(i)e\cos(4.24077-2M-\omega-3\Omega+3\theta) \\
+ & \; 1.28882 \cdot 10^{-8}e\cos(4.24077+2M+\omega-3\Omega+3\theta)-1.28882\cdot 10^{-8}\cos(i)e\cos(4.24077+2M+\omega-3\Omega+3\theta) \\
- & \; 1.28882 \cdot 10^{-8}\cos^2(i)e\cos(4.24077+2M+\omega-3\Omega+3\theta)+1.28882\cdot 10^{-8}\cos^3(i)e\cos(4.24077+2M+\omega-3\Omega+3\theta) \\
+ & \; 2.84616 \cdot 10^{-8}e^2\cos(4.24077+3M+\omega-3\Omega+3\theta)-2.84616\cdot 10^{-8}\cos(i)e^2\cos(4.24077+3M+\omega-3\Omega+3\theta) \\
- & \; 2.84616 \cdot 10^{-8}\cos^2(i)e^2\cos(4.24077+3M+\omega-3\Omega+3\theta)+2.84616\cdot 10^{-8}\cos^3(i)e^2\cos(4.24077+3M+\omega-3\Omega+3\theta) \\
+ & \; 2.27334 \cdot 10^{-8}e^2\cos(4.24077+5M+3\omega-3\Omega+3\theta)-6.82003\cdot 10^{-8}\cos(i)e^2\cos(4.24077+5M+3\omega-3\Omega+3\theta) \\
+ & \; 6.82003 \cdot 10^{-8}\cos^2(i)e^2\cos(4.24077+5M+3\omega-3\Omega+3\theta)-2.27334\cdot 10^{-8}\cos^3(i)e^2\cos(4.24077+5M+3\omega-3\Omega+3\theta) \\
- & \; 1.80186 \cdot 10^{-8}\sin(i)e^2\sin(M-\omega)+2.25232\cdot 10^{-8}(\sin(i))^3e^2\sin(M-\omega)+1.31044\cdot 10^{-8}\sin(i)\sin(M+\omega) \\
+ & \; -1.63805 \cdot 10^{-8}(\sin(i))^3\sin(M+\omega)+2.62088\cdot 10^{-8}\sin(i)e^2\sin(M+\omega)-3.2761\cdot 10^{-8}(\sin(i))^3e^2\sin(M+\omega) \\
+ & \; 3.93132 \cdot 10^{-8}\sin(i)e\sin(2M+\omega)-4.91415\cdot 10^{-8}(\sin(i))^3e\sin(2M+\omega) \\
+ & \; 8.68167 \cdot 10^{-8}\sin(i)e^2\sin(3M+\omega)-1.08521\cdot 10^{-7}(\sin(i))^3e^2\sin(3M+\omega) \\
+ & \; 2.73009 \cdot 10^{-8}(\sin(i))^3e\sin(4M+3\omega)+8.66802\cdot 10^{-8}(\sin(i))^3e^2\sin(5M+3\omega) \\
- & \; 3.84504 \cdot 10^{-8}\sin(i)e^2\sin(2.54159-5M-3\omega-2\Omega+2\theta)-7.69008\cdot 10^{-8}\cos(i)\sin(i)e^2\sin(2.54159-5M-3\omega-2\Omega+2\theta) \\
- & \; 3.84504 \cdot 10^{-8}\cos^2(i)\sin(i)e^2\sin(2.54159-5M-3\omega-2\Omega+2\theta)-1.21104\cdot 10^{-8}\sin(i)e\sin(2.54159-4M-3\omega-2\Omega+2\theta) \\
- & \; 2.42207 \cdot 10^{-8}\cos(i)\sin(i)e\sin(2.54159-4M-3\omega-2\Omega+2\theta) \\
- & \; 1.21104 \cdot 10^{-8}\cos^2(i)\sin(i)e\sin(2.54159-4M-3\omega-2\Omega+2\theta) \\
- & \; 1.60462 \cdot 10^{-8}\sin(i)e^2\sin(2.54159-3M-\omega-2\Omega+2\theta)+3.20925\cdot 10^{-8}\cos(i)\sin(i)e^2\sin(2.54159-3M-\omega-2\Omega+2\theta) \\
+ & \; 4.81387 \cdot 10^{-8}\cos^2(i)\sin(i)e^2\sin(2.54159-3M-\omega-2\Omega+2\theta)+1.60462\cdot 10^{-8}\sin(i)e^2\sin(2.54159+3M+\omega-2\Omega+2\theta) \\
+ & \; 3.20925 \cdot 10^{-8}\cos(i)\sin(i)e^2\sin(2.54159+3M+\omega-2\Omega+2\theta) \\
- & \; 4.81387 \cdot 10^{-8}\cos^2(i)\sin(i)e^2\sin(2.54159+3M+\omega-2\Omega+2\theta) \\
+ & \; 2.42207 \cdot 10^{-9}\sin(i)\sin(2.54159+3M+3\omega-2\Omega+2\theta)-4.84415\cdot 10^{-9}\cos(i)\sin(i)\sin(2.54159+3M+3\omega-2\Omega+2\theta) \\
+ & \; 2.42207 \cdot 10^{-9}\cos^2(i)\sin(i)\sin(2.54159+3M+3\omega-2\Omega+2\theta)-1.45324\cdot 10^{-8}\sin(i)e^2\sin(2.54159+3M+3\omega-2\Omega+2\theta) \\
+ & \; 2.90649 \cdot 10^{-8}\cos(i)\sin(i)e^2\sin(2.54159+3M+3\omega-2\Omega+2\theta) \\
- & \; 1.45324 \cdot 10^{-8}\cos^2(i)\sin(i)e^2\sin(2.54159+3M+3\omega-2\Omega+2\theta) \\
+ & \; 1.21104 \cdot 10^{-8}\sin(i)e\sin(2.54159+4M+3\omega-2\Omega+2\theta)-2.42207\cdot 10^{-8}\cos(i)\sin(i)e\sin(2.54159+4M+3\omega-2\Omega+2\theta) \\
+ & \; 1.21104 \cdot 10^{-8}\cos^2(i)\sin(i)e\sin(2.54159+4M+3\omega-2\Omega+2\theta)+3.84504\cdot 10^{-8}\sin(i)e^2\sin(2.54159+5M+3\omega-2\Omega+2\theta) \\
- & \; 7.69008 \cdot 10^{-8}\cos(i)\sin(i)e^2\sin(2.54159+5M+3\omega-2\Omega+2\theta) \\
+ & \; 3.84504 \cdot 10^{-8}\cos^2(i)\sin(i)e^2\sin(2.54159+5M+3\omega-2\Omega+2\theta)) \,.
\end{aligned}
$$

`Mathematica`© functions for normalization procedure

We provide here the main functions (written in `Mathematica`© language) used in the computation of the proper elements. The computation of the Poisson bracket has as input the functions involved H, χ, the variables a, m, the book-keeping parameter λ and the actual order of normalization r.

```
poissonBracketComp[H_, χ_, a_, m_, λ_, r_]:=Module[{nvars = Length[a]},
ss = 0;
chi = PowerExpand[Coefficient[χ, λ, r]];
For[j = 0, j ≤ r, j++,
      Hr = Chop[Expand[PowerExpand[Coefficient[H, λ, j]]]];
      ss = λ^(j+r) Expand[Σ_{i=1}^{nvars} ( (∂Hr/∂a[[i]]) (∂chi/∂m[[i]]) − (∂chi/∂a[[i]]) (∂Hr/∂m[[i]]) )] + ss;
];
ss];
```

The Lie series expansion is computed by using the Poisson bracket determined above, and it needs a supplementary input parameter which is the maximum Taylor order T of the expansion.

```
expLie[H_, χ_, a_, m_, T_, λ_, r_]:=Module[{},
oldH = poissonBracketComp[H, χ, a, m, λ, r];
s = H;
For[k = 1, k ≤ T, k++,
      s = Chop[Expand[PowerExpand[TrigReduce[oldH/k! + s]]]];
      newH = poissonBracketComp[oldH, χ, a, m, λ, r];
      oldH = newH;
];
Chop[s]];
```

The iterative procedure to obtain the normal form of the Hamiltonian and the associated generating functions is described in the following code.

```
For[r = 1, r ≤ maxSteps, r++,
    ℛ = Chop[Coefficient[ℋ_old, λ, r]]
    ℛ_noAngles = Chop [ℛ/. e^x_ → 0]
    ℛ_angles = TrigReduce[TrigToExp[ℛ − ℛ_noAngles]]
    For[j = 1, j ≤ Length[ℛ_noAngles], j++,
       coefℛexp = ConstantArray[Null, Length[angles]]
       For[k = 1, k ≤ Length[angles], k++,
          coefℛexp[[k]] = Coefficient[Expand[Exponent[ℛ_noAngles[[j]], e]/i], angles[[k]]];
       ];
       χ = χ + If[coef=!=nullVect ∧ |coefℛexp ∗ coefZ_0| > eps, ℛ_angles[[j]]/(i ∗ coefℛexp ∗ coefZ_0), 0];
    ];
    ℋ_new = Collect [expLie(ℋ_Old, χ, angles, momenta, maxTaylor, λ, r), λ^_] ;
    ℋ_old = ℋ_new/. λ^b_/;b > r + maxR → 0;
  ];
```

where *maxSteps* is the maximum normalization order, *angles* and *momenta* are the conjugated variables, *nullVect* is a predefined null vector, $\mathscr{H}_{\mathrm{old}} = Z_0 + \mathscr{R}_0$, with $Z_0 = angles \cdot \mathrm{coef}Z_0$ (initial linear part), and $\mathscr{R}_0$ is the initial remainder.

A sample fragment from Ariane 44lp

We report below the data for the case study Ariane 44lp (see $http://stuffin.space/TLE.json$ and $http://www.space-track.org/$). Table 1 provides the orbital elements for an overall number of 35 fragments.

Nr. of the fragment	Semimajor axis	eccentricity	inclination	argument of perigee	longitude ascending node	mean anomaly	epoch
1	17904.0191487229	0.6184822	8.4451	354.2791	29.6916	91.1478	21.3170194016986
2	18399.0276416196	0.6349184	5.6191	336.851	91.3123	304.4155	21.3158598180548
3	20543.3569281469	0.6712543	7.6605	26.3522	255.3261	358.9355	21.3145943863836
4	20626.7894213185	0.6715137	5.2669	14.9811	282.4857	218.0752	21.3084380976712
5	20786.5843639873	0.6638947	7.4939	72.3148	349.4196	158.1034	21.316183036137
6	20868.7278613271	0.6654186	7.4885	226.7365	169.4096	246.15	21.3171395796164
7	20876.2538908806	0.6765078	6.8856	358.7607	8.0042	271.7151	21.3167227489041
8	20892.9764860537	0.6813021	10.0007	306.5008	134.92	106.8055	21.3170399032877
9	21084.0476415117	0.6682642	4.1035	353.3243	105.4478	324.6696	21.3170109013425
10	21441.333004893	0.644481	7.8536	65.6596	27.3854	329.8402	21.3174512389589
11	21769.9517086512	0.688277	7.6007	4.4542	328.0948	328.1264	21.3159735364384
12	21917.0938408338	0.6919246	8.6943	306.4859	136.0569	40.5123	21.3158622749863
13	21920.2323274394	0.6870708	9.3124	328.6102	114.8056	44.4079	21.3156164263836
14	22064.4052364507	0.6929217	6.929	45.6923	229.817	154.3189	21.3165683189863
15	22917.2281907394	0.7065298	6.9189	13.4068	279.5081	171.4635	21.3165301910137
16	23630.6459758059	0.7058464	6.9866	349.079	71.4477	100.7221	21.3023051550137
17	23752.7576596151	0.7067865	6.5391	355.4221	35.32	259.7166	21.3161448218904
18	23777.9969111247	0.7107788	6.5247	329.9817	116.9968	216.3228	21.3163193070959
19	23807.8276319183	0.7157412	6.5825	8.9343	295.6527	126.3599	21.3161221366027
20	23869.3235909869	0.7183124	6.6369	5.5882	314.3596	115.9273	21.3157300567123
21	24060.6709580804	0.7049004	6.8932	235.1089	14.3698	318.4293	21.3172805911233
22	24113.961510087	0.7165558	6.4841	342.8896	94.3393	227.9558	21.3152282643836
23	24274.1586562043	0.7190037	6.6569	356.2004	31.5032	261.1107	21.3164713207123
24	24544.3718942564	0.716221	7.3948	353.282	92.7314	46.693	21.3173799029863
25	24660.0785514902	0.7124817	6.7994	112.4036	209.3436	169.3944	21.3168953355342
26	24680.3948970579	0.7095337	6.392	5.9843	92.3051	233.2283	21.3169514506301
27	24724.0341006982	0.7116403	6.5975	278.1543	150.9718	200.4893	21.3169310602466
28	24785.1181300407	0.7212263	6.7212	269.8277	341.7454	104.035	21.3171603729041
29	24846.9547189182	0.7008588	6.7947	43.2835	62.4869	71.5338	21.3166421711507
30	24877.4771968171	0.7208086	6.9462	279.3927	106.5504	306.1258	21.3163118204384
31	25260.9137486811	0.7138288	7.1158	7.303	171.4194	15.6608	21.3105989725753
32	25276.9066136242	0.7043189	7.4881	161.2966	3.828	89.9672	21.3173322156712
33	25414.6566259262	0.7207708	6.9084	13.2566	268.9859	329.8468	21.3166764046849
34	25609.6309977115	0.7138026	8.6906	8.0081	302.3345	18.8057	21.3117860994795
35	25687.2108421546	0.715049	8.7177	118.8734	195.3687	67.8641	21.3067049292055

Table 1. Orbital elements of the fragments associated to Ariane 44lp.

We select fragment nr. 6 in Table 1 for which we provide below the (i) osculating elements at time $t = 0$, (ii) the osculating elements at $t = 150$ years, (iii) the normal form, (iv) the analytic solution, and (v) the proper elements.

(i) Osculating elements at time $t = 0$: $a = 20868.7$, $e = 0.665419$, $i = 7.4885^o$, $\omega = 246.15^o$, $\Omega = 226.737^o$, $M = 169.41$.

(ii) Osculating elements at $t = 150$ years: $a = 20867.3$, $e = 0.665796$, $i = 7.59732^o$, $\omega = 236.0826531^o$, $\Omega = 271.2911684^o$, $M = 236.8937408^o$.

(iii) The normal form is given by the following expression:

$$\begin{aligned} K(P,Q) &= 0.0790084P^3 + P^2(-0.0170398 - 0.0756912Q) - 0.00139873Q - 0.00134323Q^2 \\ &+ P(0.00275497 + 0.0132787Q + 0.0127518Q^2) - 0.000146798Q_M \; . \end{aligned}$$

(iv) We provide the analytic solution for the eccentricity, obtained back-transforming from the normal form elements to the original elements:

$$\begin{aligned} e(t) &= \Big((-6.31238 \cdot 10^{-8})\cos(3.21433t) - (4.89905 \cdot 10^{-6})\cos(5.88655t) \\ &- (9.97053 \cdot 10^{-5})\cos(6.22367t) + (3.64676 \cdot 10^{-5})\cos(6.32616t) \\ &- (1.41399 \cdot 10^{-4})\cos(6.56079t) - (1.73199 \cdot 10^{-5})\cos(6.89791t) \\ &- (2.68138 \cdot 10^{-6})\cos(9.10087t) - (6.10999 \cdot 10^{-5})\cos(9.438t) \\ &- (2.82243 \cdot 10^{-5})\cos(9.77512t) + (6.43298 \cdot 10^{-7})\cos((1.01122 \cdot 10)t) \end{aligned}$$

$$
\begin{aligned}
&- (5.35938\cdot 10^{-7})\cos((1.23152\cdot 10)t) + (7.29325\cdot 10^{-8})\cos((1.24473\cdot 10)t)\\
&- (8.48576\cdot 10^{-6})\cos((1.26523\cdot 10)t) + (1.01882\cdot 10^{-6})\cos((1.29894\cdot 10)t)\\
&+ (5.76535\cdot 10^{-8})\cos((1.56617\cdot 10)t) + (2.69513\cdot 10^{-7})\cos((1.58666\cdot 10)t)\\
&- (5.46793\cdot 10^{-9})\sin(3.21433t) - (6.46828\cdot 10^{-6})\sin(5.88655t)\\
&+ (5.78642\cdot 10^{-4})\sin(6.22367t) + (5.1771\cdot 10^{-5})\sin(6.32616t)\\
&+ (9.29051\cdot 10^{-5})\sin(6.56079t) - (4.19333\cdot 10^{-6})\sin(6.89791t)\\
&- (4.2597\cdot 10^{-6})\sin(9.10087t) + (2.32447\cdot 10^{-4})\sin(9.438t)\\
&+ (1.52328\cdot 10^{-5})\sin(9.77512t) + (2.16004\cdot 10^{-7})\sin((1.01122\cdot 10)t)\\
&- (1.04109\cdot 10^{-6})\sin((1.23152\cdot 10)t) + (2.59029\cdot 10^{-8})\sin((1.24473\cdot 10)t)\\
&+ (2.37284\cdot 10^{-5})\sin((1.26523\cdot 10)t) - (4.40994\cdot 10^{-7})\sin((1.29894\cdot 10)t)\\
&+ (2.6279\cdot 10^{-8})\sin((1.56617\cdot 10)t) - (5.87886\cdot 10^{-7})\sin((1.58666\cdot 10)t) + 4.43174\cdot 10^{-1}\Big)^{\frac{1}{2}} .
\end{aligned}
$$

Below we provide the analytic solution for the inclination, obtained back-transforming from the normal form elements to the original elements:

$$
i(t) = \frac{180^o}{\pi}\ \arccos\frac{\alpha}{\beta}\ ,
$$

where

$$
\begin{aligned}
\alpha \;=\;& -\cos(5.22865 - (1.9081\cdot 10)t) - (7.58533\cdot 10^{2})\cos(2.00065 - (1.58666\cdot 10)t)\\
&- 8.86657\cos(5.77305\cdot 10 - (1.55295\cdot 10)t) + (5.9036\cdot 10^{3})\cos(5.12745\cdot 10 - 9.10087t)\\
&- 1.12801\cos(1.07004\cdot 10^{2} - 8.76375t) - (1.8148\cdot 10^{4})\cos(6.456 - 6.42865t)\\
&- (5.8175\cdot 10^{3})\cos(6.21858\cdot 10 - 6.09153t) - (1.9034\cdot 10^{4})\cos(4.80465\cdot 10 - 5.88655t)\\
&- (3.79396\cdot 10)\cos(1.17916\cdot 10^{2} - 5.75441t) - (1.23935\cdot 10^{6})\cos(3.228 - 3.21433t)\\
&- (1.91375\cdot 10^{5})\cos(5.89578\cdot 10 - 2.8772t) + (4.74512\cdot 10^{3})\cos(1.14688\cdot 10^{2} - 2.54008t)\\
&+ (2.26509\cdot 10^{4})\cos(3.55145t + 5.25018\cdot 10) - 3.71603\cos(3.88857t + 1.08232\cdot 10^{2})\\
&+ (1.37737\cdot 10^{6})\cos(6.22367t + 7.68335) + (3.96881\cdot 10^{5})\cos(6.56079t + 6.34132\cdot 10)\\
&+ (1.25389\cdot 10)\cos(6.76577t + 4.92738\cdot 10) + (4.18025\cdot 10^{4})\cos(6.89791t + 1.19143\cdot 10^{2})\\
&- (2.81897\cdot 10^{5})\cos(9.438t + 4.45535) - (3.76177\cdot 10^{4})\cos(9.77512t + 6.01852\cdot 10)\\
&+ (7.95918\cdot 10^{2})\cos((1.01122\cdot 10)t + 1.15915\cdot 10^{2})\\
&+ 1.24163\cos((1.62038\cdot 10)t + 5.37292\cdot 10) + 2.58887\cdot 10^{9}\ ,
\end{aligned}
$$

$$
\begin{aligned}
\beta \;=\;& \cos(5.22865 - (1.9081\cdot 10)t) + (1.51707\cdot 10^{3})\cos(2.00065 - (1.58666\cdot 10)t)\\
&+ (1.77331\cdot 10)\cos(5.77305\cdot 10 - (1.55295\cdot 10)t)\\
&- (2.74678\cdot 10^{3})\cos(5.45025\cdot 10 - (1.23152\cdot 10)t)\\
&+ 1.57647\cos(1.10232\cdot 10^{2} - (1.19781\cdot 10)t) + (1.18072\cdot 10^{4})\cos(5.12745\cdot 10 - 9.10087t)\\
&- 2.25603\cos(1.07004\cdot 10^{2} - 8.76375t) - (1.9034\cdot 10^{4})\cos(4.80465\cdot 10 - 5.88655t)\\
&+ (1.37737\cdot 10^{6})\cos(6.22367t + 7.68335) + (3.96881\cdot 10^{5})\cos(6.56079t + 6.34132\cdot 10)\\
&+ (4.18025\cdot 10^{4})\cos(6.89791t + 1.19143\cdot 10^{2}) - (5.63794\cdot 10^{5})\cos(9.438t + 4.45535)\\
&- (7.52354\cdot 10^{4})\cos(9.77512t + 6.01852\cdot 10) + (1.59184\cdot 10^{3})\cos((1.01122\cdot 10)t + 1.15915\cdot 10^{2})\\
&+ (5.91141\cdot 10^{4})\cos((1.26523\cdot 10)t + 1.22735) - (2.60421\cdot 10^{3})\cos((1.29894\cdot 10)t\\
&+ 5.69572\cdot 10) + 1.41697\cos((1.33266\cdot 10)t + 1.12687\cdot 10^{2})\\
&- 2.48327\cos((1.62038\cdot 10)t + 5.37292\cdot 10)\\
&- (1.48548\cdot 10^{5})\sin(6.32616t + 6.13675\cdot 10^{-1}) + 2.61238\cdot 10^{9}\ .
\end{aligned}
$$

(v) The values of the proper elements are the following: the proper eccentricity is 0.665715 and the proper inclination is 7.69522.
